# COVID-19: An International Public Health Concern

**DOI:** 10.5195/cajgh.2020.466

**Published:** 2020-04-01

**Authors:** Israel Oluwasegun Ayenigbara

**Affiliations:** 1 Department of Health Education, School and Community Health Unit, University of Ibadan, Ibadan, Nigeria

**Keywords:** COVID-19 infection, reservoir of infection, mode of transmission, signs and symptoms, preventive measures

## Abstract

This review presents a synopsis on the current COVID-19 pandemic, with focus on preventive measures. COVID-19 is a new viral infection, and is in form of a positive-sense, single-stranded RNA Coronavirus which belongs to an expanded group of viruses which were identified six decades ago. Importantly, the new COVID-19 belongs to the group of SARS-CoV, and it originated in bats but infected humans through smuggled pangolins. At first, the mode of transmission of infection was animal-to-person, but person-to-person and community transmission of the virus has been confirmed in many parts of the world. With an incubation period of between two-fourteen days, signs and symptoms of infection are mild to high respiratory illness; characterized with cough, breathing problems (shortness of breath), high temperature (Fever), tiredness (Fatigue) and nausea. Presently, no vaccines or specific treatment is available for COVID-19, in light of the aforementioned; prevention is the only substantial and less expensive option. With the envisaged explosive community transmission of COVID-19 in the coming weeks in places with limited daily testing, especially in African countries, it is recommended among many that social distancing which includes avoiding any form of contact with people; either through greetings, hugging or shaking of hands and large gatherings, avoid contact with animal items, dead or alive animals, sick and dead people from areas experiencing COVID-19 epidemic, and basic hygienic practices like thorough washing of hands with clean water and antiseptic soap for the duration of at least twenty seconds should be practiced always. However, in the absence of the aforementioned, an alcohol-based hand gel should be used on the hands frequently. Furthermore, health care workers should adhere strictly to the standard preventive measures in areas of heightened COVID-19 epidemic.

## Introductions

A recently discovered coronavirus named officially as “COVID-19” is currently spreading at a fast rate in China, and numerous cases of the new infection has now been confirmed in many countries as well. With the continuous upsurge in the number of laboratory confirmed cases and mortality, scientists, physicians and health care workers around the world are working assiduously to know more about the epidemiology of the new virus, so as to enable appropriate measures to be put in place to forestall and limit its rapid spread [1, 2]. Generally, Coronaviruses belongs to an expanded family of viruses which were identified exactly six (6) decades ago [2]. The first sets of viruses which belong to the Coronaviruses family were indentified in chicken (infectious bronchitis virus) and in humans, namely human coronavirus 229E and human coronavirus OC43; symptoms manifest as common cold [3]. Over the years, numerous members of the Coronaviruses family were discovered, they are SARS-CoV (2003), HCoV NL63 (2004), HKU1 (2005), MERS-CoV (2012), and the current COVID-19 pandemic which was discovered at the end of 2019; majority of these viruses had resulted in the severe infections of the respiratory system, and some causes sickness in people [3, 4]. In addition, many other coronaviruses affect animals like cats, bats and camels [4]. Importantly, many individuals are infected with coronaviruses during their lifetime, but signs and symptoms are usually not severe, although, mild pneumonia can result in some cases [2].

The Novel COVID-19 pandemic is a positive-sense, single-stranded RNA coronavirus [5, 6, 7]. The World Health Organization (WHO) was alerted of the first suspected cases of the infection in late 2019 (31^st^ December 2019), which was just over three weeks after the manifestation of signs and symptoms in the suspected cases [8, 9]. More findings of the new COVID-19 infection revealed that the novel virus was genomically identified in a sample from a person with pneumonia in Wuhan Province, China [10]. Although, this current COVID-19 pandemic is unprecedented, and scientist, researchers and health workers around the world are still getting to know more about its epidemiology in entirety. With the increase in the number of confirmed cases and deaths been reported every day in the world, the current situation is now a pandemic. Hence, this review paper gives a synopsis of the current COVID-19 pandemic under its probable Reservoir Species, Mode of transmission, Signs and Symptoms, Current situation around the world, Africa and Nigeria, and importantly, Preventive measures to be adopted to forestall the spread of the pandemic.

## Sources of Information

This is a review research on COVID-19 pandemic, and discussion were outlined under probable Reservoir Species, Mode of transmission, Signs and Symptoms, Current situation around the world, the African continent and Nigeria, and importantly and Preventive measures were provided. Sources of materials for the review were gotten from the various international and national health authorities such as the World Health Organization and the US Centers for Disease Control and Prevention publications. Furthermore, major scientific documents included in this review were also gotten from PubMed databases.

## Reservoir Species

The new COVID-19 belongs to the group of SARS-CoV. In 2015 and 2017, two genome sequences from Rhinolophus sinicus with a resemblance of 80% had been published; the third unpublished virus genome from Rhinolophus affinis with a resemblance of 96% to the current COVID-19 pandemic was mentioned in an article from the Wuhan Institute of Virology [11, 12]. For comparison, this sequence of mutation is the same to the ones seen over a decade ago in the H3N2 human flu epidemic [11, 13]. Furthermore, pairwise protein sequence analysis of seven conserved non-structural proteins domains proves that COVID-19 belongs to the species of SARSr-CoV. In addition, the virus causing the current COVID-19 pandemic was isolated from the bronchoalveolar lavage fluid of a critically ill patient; the virus uses the same cell entry receptor-angiotensin converting enzyme II (ACE2) as that of SARS-CoV [14, 15, 16]. Animals sold as human food consumption are the first source of transmission of the current COVID-19 outbreak due to the fact that majority of the first sets of human causalities noted and confirmed infected cases were workers at the Huanan animal Market in Wuhan province, before exposure to larger contacts of humans and animals [17].

## Mode of Transmission

The first sets of patients from the COVID-19 outbreak in Wuhan province, China were workers or buyers at the main animal market in the province, indicating transmission through animal-to-human [6, 18]. Importantly, there has been an upsurge in the number of sick individuals from the COVID-19 who have not had exposure or contact to the Wuhan main animal markets whatsoever, this indicates that human-to-human and community transmission of COVID-19 is happening [6, 19, 20, 21]. Specifically, person-to-person spread of the COVID-19 outbreak was affirmed on 20^th^ January 2020 in Guangdong, China, by the Chinese authority [22]. It is now known how the human-to-human transmission of the current COVID-19 pandemic occurred, for instance, when person-to-person transmission occurred with Middle East Respiratory Syndrome (MERS) and Severe Acute Respiratory Syndrome (SARS) infections happened, it occurred through respiratory droplets from sick patient's, and infected coughs or sneezes from sick patients, the same way many respiratory pathogens circulates to humans, and the human-to-human transmission generally occurred between close contacts [6]. Importantly, human-to-human transmission of viruses varies, some viruses have high virulence and are highly contagious, while other viruses are less so [6]. The person-to-person spread of the current COVID-19 pandemic is primarily spread between people via respiratory droplets from coughs and sneezes [23, 24]. As of 29^th^ February 2020, community transmission of COVID-19 pandemic has been confirmed in China, Germany, Hong Kong, Iran, Italy, Spain, France, Japan, Singapore, South Korea and the United States of America [25, 26]. Furthermore, there is a possibility that the current COVID-19 pandemic might be spread through fecal oral route of transmission as a healthy six-month-old baby with COVID-19 had persistently positive nasopharyngeal swabs to day sixteen (16) of admission [27]. Firstly, this scenario pinpoints the task in establishing the exact incidence of COVID-19 transmission as asymptomatic individuals can excrete the virus which can pose a major threats in areas where there are not good drinking water supply, e.g. in the developing countries. Secondly, these set of individuals can be the causative link to some undetected transmission of the virus in the community [27].

## Signs and Symptoms

The time for the manifestation of signs and symptoms of COVID-19 is between two to fourteen days, as this was the time range the signs and symptoms began to appear after an exposure to MERS virus [28]. Although, some studies caution that the average incubation period of COVID-19 is between six and half days, and ranges from zero to twenty-four days, and the basic reproductive number (R0) of the pandemic is between 2 to 3.5 at the early phase regardless of different prediction models, which presently in comparison, is more than SARS and MERS [29]. Specifically, individuals with confirmed cases of COVID-19 have signs and symptoms of low to high respiratory illness; characterized with cough, breathing problems (shortness of breath), high temperature (Fever), tiredness (Fatigue) and nausea [17, 30, 31, 32]. Furthermore, chronic inflammation of the lung (pneumonia), malfunctioning of the kidney (kidney failure) and death have also appeared in patients with severe cases of COVID-19 [33, 34, 35, 36]. Importantly, in hospitalized individuals, specific vital signs parameters such as body temperature, blood pressure, pulse (heart rate) and breathing rate (respiratory rate) were stable during admission, but infected patients had an abnormally low count of leukocytes (leucopenia) and abnormally reduced number lymphocytes in their blood (lymphopenia) [17, 37]. Also, few numbers of individuals with COVID-19 experienced serious signs and symptoms, and majority of hospitalized patients have one or two fundamental health conditions such as diabetes, cardiovascular diseases and hypertension [38] (Table 1).

## Current Situation of COVID-19 Infection in the World, Africa, and Nigeria

The WHO was notified of numerous cases of pneumonia on 31^st^ December 2019, specifically in Wuhan, Hubei Province of China [41]. After preliminary investigation, the virus did not match any existing viruses, and this heightened concerns among scientists because the route of transmission was not known since it is a new virus [41]. On 7^th^ January, approximately a week later, the Chinese government confirmed they had identified the new virus, and it is a coronavirus which belongs to the group of viruses that include the common cold, and viruses such as SARS and MERS [41]. With the evolving nature of this current pandemic, and the frequent changes in statistics and figures, as at 29^th^ March 2020, there have been 697,994 confirmed cases of COVID-19 from 202 countries and territories and one conveyance ship, and 33,421 deaths recorded, with majority of the world's population on lockdown to avert the further spread of the infection [42]. Explicitly, the Western Pacific Region (Nineteen countries and territories affected) has a total number of 104,146 confirmed cases, with 3,660 deaths; the European Region (Sixty countries and territories affected) has a total number of 397,719 confirmed cases, with 24,246 deaths, the South-East Asia Region (Ten countries affected) has a total number of 4,333 confirmed cases, with 164 deaths, the Eastern Mediterranean Region (Twenty-one countries and territories affected) has total number of 46,623 confirmed cases, with 2,828 deaths, the Region of the Americas (Fifty-one countries and territories affected) has a total number of 141,282 confirmed cases, with 2,461 deaths, the African Region (Forty-one countries and territories affected) has a total number of 3,179 confirmed cases, with 55 deaths, while the Conveyance Diamond Princess ship has a total number of 712 confirmed cases, with 7 deaths [42]. Presently, the European Region with Sixty countries and territories affected with a total number of 397,719 confirmed cases and 24,246 deaths have now overtaken the Western Pacific Region which included China as the current epicenter of the current COVID-19 pandemic [42]. The African region with Forty-one countries and territories affected has the lowest number of confirmed cases and deaths with 3,179 and 55 respectively. However, massive sensitization and heightened preventive measures which includes rigorous contact tracing and wider testing should be intensified so as to prevent against explosive community transmission and mortalities of COVID-19 due to the weak health care system in the African continent [42]. Initially, the majority of confirmed cases of COVID-19 has been in China where the outbreak started, however, massive COVID-19 cases and mortalities have occurred in other countries as well, for instance in the United States of America, Italy, Iran, France, Britain, France and Spain [43, 44]. The exact number of individuals who have contracted the virus could be far higher as people with mild symptoms are not been detected due to shortages in test kits, especially in developing countries [44].

The COVID-19 Pandemic spread to the Africa continent on 14^th^ February 2020, with Egypt recording the first confirmed case of the virus on the continent, while the first recorded case in the sub-Saharan Africa was in Nigeria; both cases were imported transmission [45, 46, 47]. Afterwards, subsequent cases of COVID-19 have come from imported cases from Europe and the United States rather than from China; the country where the infection broke out [48]. Presently, according to the WHO classification of regions, Forty-one Africa countries and territories have reported a total number of 3,179 confirmed cases and 55 deaths from COVID-19 pandemic, these figures are expected to rise in the coming weeks [42]. Also, confirmed cases of infection and deaths from COVID-19 pandemic have been reported in some African countries (Egypt, Morocco, Tunisia, Sudan, Libya and Somalia) which are classified in the Eastern Mediterranean region by the WHO [42]. As at 28^th^ March 2020, Botswana, Burundi, Comoros, Lesotho, Malawi, Sao Tome and Principe, Sierra Leone, and South Sudan remains the only African countries who have yet to report any cases of COVID-19. Importantly, numerous preventive measures have been implemented to abate the spread of COVID-19 in different countries in Africa, these include travel restrictions, school closures, border closures, flights and event cancellations [49, 50]. Although, explosive community transmission of the infection is predicted in the African continent due to limited testing and inadequate contact tracing, however, the experience gathered during the Ebola and Lassa fever epidemics could help in the continents battle to contain the COVID-19 pandemic [49].

In Nigeria, with the rapid changes in infection statistics, a total of 111 confirmed cases and a single death have been recorded, with three patients discharged after treatment from COVID-19 as at 29th of March 2020, and confirmed cases have mild to moderate signs and symptoms that are currently receiving care [51]. In all, Lagos state has the highest number of confirmed cases in Nigeria with sixty-eight patients, followed by Abuja, Oyo, Ogun, Enugu, Bauchi, Osun, Edo, Benue, Ekiti, Kaduna and Rivers with combined confirmed cases of forty-three [51]. Unfortunately, limited COVID-19 tests are been done in Nigeria due to shortages of test kits, hence, the number of infection cases due to community transmission is predicted to increase sharply in the coming weeks. Although, as precautionary measures to avert the rate of infection, the president ordered the cessation of all none essential movements in Lagos and the capital Abuja; the two cities with the highest number of confirmed cases for an upward reviewable duration of fourteen days which starts on Monday, 30th March 2020 [52]. Importantly, Several countries in the world have also put up various preventive measures to curtail the spread of COVID-19, some of which are travel bans to and from the countries that are massively affected, compulsory quarantine, lockdown of cities, restricted access to some specific places, compulsory checking at borders, airports and railway terminals, and presently, the Chinese authorities have stopped and banned wildlife trade nationwide, at least until the current pandemic has ended, as this was believed to be the source of the current COVID-19 pandemic and the SARS epidemic almost twenty years ago [44].

## Prevention of COVID-19 Infection

Presently, no vaccines or specific treatment is available for COVID-19, but there are ongoing studies on preventive vaccines, and some drugs are currently been used for the treatment of this current pandemic [53, 54, 55, 56, 57, 58, 59]. In light of the aforementioned, prevention is the only substantial option against COVID-19. Firstly, if traveling to locations where there are confirmed cases of COVID-19 outbreak, it is pertinent to avoid any form of contact either through greetings, hugging, kissing or shaking of hands. Also, contact with sick people, items that come from animals such as uncooked meat should be ultimately avoided [2, 60]. Furthermore, basic hygienic practices like thorough washing of hands with clean water and antiseptic soap for the duration of twenty seconds should be practiced always. However, in the absence of the aforementioned, an alcohol-based hand gel should be used on the hands frequently [60]. As recommended by the CDC and WHO; contact with unwashed hands should be avoided on the nose, mouth, eyes and the ears. Furthermore, reduce and minimize close contact with people, especially sick individuals, self isolation when sick, fourteen days compulsory quarantine after returning from locations that have recorded cases of the COVID-19, cough and sneeze on a tissue paper, afterwards dispose the tissue paper in a waste bin, clean and disinfect frequently touched objects and surfaces; all these are imperative preventive measures to be adopted in the prevention of the current COVID-19 [42, 61].

For health workers, Infection control training should be to all clinical staff, installation and provision of protective shields, frequent disinfection of equipment, and provision of eye protective equipment should be provided to all medical staff treating COVID-19 patients [62]. Also, as precautionary, medical workers should measure their own body temperatures before and after work, and promptly report any symptoms of upper respiratory tract infection, vomiting or diarrhea [62]. Furthermore, universal masking, hand hygiene, and appropriate use of personal protective equipment (PPE) should be well implemented by medical workers [63]. In addition, medical staff should be extremely careful with blood samples collected for diagnosis, as there is still a risk of transmission of COVID-19 virus through the transfusion of labile blood products, as more and more asymptomatic infections are being found among the current COVID-19 cases [63]. Also, to promptly identify patients and prevent further spreading of infection, physicians should be aware of the travel or contact history of patients with compatible COVID-19 signs and symptoms [64].

## Conclusions

COVID-19 belongs to the group of SARS-CoV, and it originated from bats, but infected humans through smuggled pangolins. At first, the mode of transmission of the current pandemic was animal-to-person, but person-to-person and community transmission of the COVID-19 has been confirmed in many parts of the world. With the continuous rise in the number of confirmed cases and mortality rates across all countries and regions in the world, category of people with the highest causalities are older people, especially those with underlying medical conditions; hence, preventive measures should be focused more on this vulnerable group. From this review, it was concluded that the COVID-19 is indeed an international public health concern due to the vast majority of countries that have reported confirmed cases of infection and deaths. However, even without the present breakthrough in the urgent search for specific treatment and vaccines, the spread of the current COVID-19 pandemic can be reduced and ultimately stopped with strict adherence and compliance to basic infection preventive measures that have been provided in this review. Furthermore, rigorous contact tracing should be implemented in African countries and in countries where limited testing has been carried as a result of shortages in test materials and equipment to prevent against the envisaged explosive community transmission of COVID-19.

## Recommendations

Based on the findings of this review, the following recommendations are further made;

1. Avoid unnecessary travel at this time, especially to areas with confirmed cases of COVID-19 outbreak.
2. Place self on compulsory fourteen days quarantine and isolation when returning from locations with the confirmed cases of COVID-19 even if there is manifestation of any signs and symptoms or not.
3. Strict testing as border points, airports and terminals should be enforced to prevent against imported cases of the COVID-19.
4. Rigorous contact tracing should be done to identify people who might have had contact with patients who are infected with COVID-19 to further prevent against the community transmission of the virus.
5. With the high numbers of causalities as a result of COVID-19 among older people, large gatherings of people, especially the elderly should be banned for now.
6. Although, there are few cases of the COVID-19 in Africa. Nevertheless, health education and sensitization programmes on preventive measures should be heightened to enlighten the public due to the vulnerable health care system in the continent.

Countries experiencing increasing number of cases from the COVID-19 should be open with their statistics, as this would help relevant health authorities to putting up appropriate preventive measures, hence helping to reducing further the number of infections.
